# Barriers and Facilitators to Accessing Healthcare for People With Parkinson's Disease in Latin America: A Qualitative Study

**DOI:** 10.1111/hex.70380

**Published:** 2025-08-13

**Authors:** Christine Jeyachandran, Catherine Spooner, Ana Margarita Rodriguez Salgado, Matthew Prina, Joel Rhee, Jorge Jesus Llibre‐Guerra, Dani Kim, Juan J. Llibre‐Rodriguez, Mark F. Harris

**Affiliations:** ^1^ The International Centre for Future Health System, UNSW Sydney Australia; ^2^ Global Brain Health Institute University of San Francisco San Francisco California USA; ^3^ Population Health Sciences Institute, Faculty of Medical Sciences Newcastle University Newcastle upon Tyne UK; ^4^ Discipline of General Practice, School of Clinical Medicine, Faculty of Medicine and Health UNSW Sydney Australia; ^5^ Department of Neurology Washington University School of Medicine in St. Louis St. Louis Missouri USA; ^6^ Health Service and Population Research Department, Institute of Psychiatry, Psychology, and Neuroscience King's College London London UK; ^7^ Dementia Research Unit Medical University of Havana Havana Cuba

**Keywords:** access to healthcare, barriers, carers, Latin America, Parkinson's disease, peer support, self‐management, stigma

## Abstract

**Aims:**

The aim of the study was to identify the barriers and facilitators to healthcare access for people with Parkinson's disease (PWP) in Spanish‐speaking Latin American countries (LAC) in Central and South America, to understand their drivers and consider the implications for health systems in LAC.

**Methods:**

Four online semi‐structured focus groups were conducted with 25 PWP who provide education and/or support to PWP. The study was designed and implemented by a person with lived experience of PD. Data were mapped to an existing model of access to healthcare that incorporates provider and consumer abilities.

**Findings:**

There were multiple provider barriers in terms of availability, affordability and appropriateness of care, driven by a lack of health system capacity. Doctors didn't recognise Parkinson's symptoms, which resulted in delayed diagnosis. Limited knowledge of Parkinson's and limited multidisciplinary care and medication was common across Spanish‐speaking Latin America. Inequities in access were experienced by those living in rural areas and those who could not afford private care. Barriers at the person level included stigma, depression and lack of health literacy around PD. Family and peer support were facilitators of access.

**Conclusion:**

Significant gaps in Parkinson's care across Latin America are driven by stigma and limited service availability. This study highlights the need for culturally tailored interventions that address stigma, promote peer support and strengthen self‐management and health professional training across Spanish‐speaking Latin America. We call for more global partnerships to encourage training and mentoring in regional cities across Latin America with co‐designed approaches to ensure a culturally appropriate framework of care that supports patients and healthcare professionals with a focus on self‐management.

**Patient or Public Contribution:**

The first author (C.J.) has Parkinson's disease (PD) and lived with the condition for 8 years in Peru. She was involved in all aspects of the study, including design, data collection, analysis and writing this article. C.J. is an international advocate in the PD community.

## 1 Introduction

Parkinson's disease (PD) is a complex neurodegenerative condition with motor symptoms, bradykinesia (slowness), trembling, rigidity and many non‐motor symptoms including apathy, depression and cognitive decline [1]. In 2021, 11.8 million people were estimated to be living with PD globally, making it the fastest‐growing neurological cause of disability [2]. Access to timely diagnosis, movement disorder specialists (MDS) and multidisciplinary care remains limited worldwide [3, 4, 5, 6]. These challenges are particularly pronounced in low‐ and middle‐income countries, including Latin American countries (LAC), where significant gaps remain in understanding access to barriers and their link to poorer outcomes [5, 7, 8, 9, 10].

Globally, PD prevalence is estimated at 315 per 100,000 and 472 per 100,000 in LAC, with the burden increasing due to rapid demographic ageing [11, 12]. In LAC, people with Parkinson's disease (PWP) experience higher rates of dependency, early mortality, psychiatric symptoms and poor mental health outcomes [10, 13, 14]. Yet, the influence of contextual and systemic barriers on these outcomes is underexplored. While stigma is a global issue among PWP—contributing to isolation, depression and reduced quality of life [6, 9, 15, 16, 17, 18, 19, 20]—little is known about its forms and impacts in Spanish‐speaking Latin America [21, 22, 23]. Generally, patients are often portrayed negatively in literature—as unmotivated, non‐adherent or lacking health literacy—while facilitators of access are rarely emphasised [3, 4, 6]. Communication from healthcare professionals (HCPs) is little documented in the region [3]. Likewise, existing studies primarily reflect provider perspectives, with limited input from PWP or peer advocates, which has been of value in the high‐income countries [24, 25]. Our study aims to give voice to the lived experience of Spanish‐speaking Latin Americans who can explain the unmet needs.

This study aims to identify barriers and facilitators to healthcare access for PWP in Spanish‐speaking LAC, using Levesque et al.'s access model [26], which defines access as the opportunity to obtain health when a need is perceived. The qualitative study uses focus groups with PWP who are peer advocates with personal experiences and knowledge of the experiences of others with Parkinson's, which will inform future research, policy and advocacy.

### 1.1 Context of Healthcare Systems

Spanish‐speaking LAC consist of 21 countries with diverse health systems, histories and economic structures [27, 28, 29] (Table 1). Many LAC have basic public health (a safety net system), but informal labour market workers in some countries cannot contribute to copayments or to tax‐based financing [30]. Public healthcare expenditure ranges from 5.6% and 9.8% of GDP (see Table 1) with significant reliance on private insurance, co‐payments and out‐of‐pocket expenditure (16%–43%) [28] (see Table 1) [27, 28].

**Table 1:** Context of healthcare systems.

Country	Population 2023	Doctors per 10,000	Out‐of‐pocket expend. As % of current expend. (2021)	Current health expenditure (% of GDP)
Argentina	46,234,830	40.8	22.40%	9.8
Chile	19,612,723	29.7	30.30%	9
Colombia	52,194,491	24.5	13.70%	7.2
Ecuador	18,511,400		30.60%	8.4
El Salvador	6,528,537	29.1	26.70%	6.2
Guatemala	19,672,073		61.00%	5.8
Mexico	130,262,216	25.6	41,4%	6.4
Peru	33,728,392	16.5	27.20%	5.6
Uruguay	3,559,917	45.1	15.40%	9.5
Venezuela	28,250,500		28.10%	6.3

*Note:* WHO Global Health Expenditure Database [29], World Bank [30], Pan American Health Organization [31] and Global Health Observatory [32].

## 2 Methods

### 2.1 Study Design and Research Question

This qualitative study utilised four online semi‐structured focus groups to explore the research question: What are the barriers and facilitators to healthcare access for PWP in Spanish‐speaking LAC?

### 2.2 Research Team

This study was led by a lived experience researcher to explore community‐based participatory research (CBPR) involving Latin American peer advocates and PWP as co‐creators in identifying health needs and shaping recommendations [31]. Focus groups were conducted online with a psychologist of Cuban ethnicity, chairing all groups while based in the United States. The first author assisted with all groups while located in Australia. The other investigators were not involved in the conduct of the focus groups, but were involved in the planning of the study, reviewed the transcripts and contributed to the analysis, interpretation and writing. The research was overseen by the University of New South Wales (UNSW) authors and supported by a team of researchers, mainly of LAC ethnicity, from the project: The epidemiology of ParkINson in LatiN AmeriCa: Learning from undEr‐represented populations (PINNACLE).

### 2.3 Recruitment and Study Participants

The inclusion criteria were 18 years or older, cognitive capacity, lived experience with PD in LAC, and engagement in peer support (advocacy, education or support), which enabled peers to provide insight into PD beyond just personal experiences.

Convenience sampling was conducted via emailing the ‘Alianza Iberoamericana de Parkinson’ mailing list (140 contacts) with an invitation to complete an expression of interest. This is a Spanish‐speaking peer‐led network of independent individuals and professionals interested in PD in LAC. Ethics requested that cognitive eligibility be confirmed, which the first author led via scripted calls on MS Teams (see Appendix 1—Call Script).

Of 32 expressions of interest, 3 were ineligible (not PD peer advocates) or did not follow through, and 25 were enrolled into four groups based on availability. We monitored diversity and the final group was recruited via follow‐up email to address the deficit in diversity, which aimed to have approximate equality and diversity of countries, genders and regional/city dwelling (etc.). A group distribution table is in Appendix 2.

Four carers (three spouses and one friend) attended in a supportive role (technical/voice clarity/emotional, etc.) at the choice of the PWP and completed carer‐specific consent forms.

The sample size was chosen to follow recommendations in the literature based on expected thematic saturation [32]. There was very little new information in the fourth discussion group so it was decided that data saturation had been reached.

### 2.4 Data Collection

Four focus groups, held between November 2023 and February 2024, were conducted in Spanish, led by a psychologist researcher (A.M.R.S.) and assisted by C.J., who has PD and who lived in Peru for the first eight years of her diagnosis. She was involved in all aspects of the study, including design, data collection, analysis and article writing. They were conducted in Spanish and recorded on Microsoft Teams, lasting approximately 1.5–2 h each. Participants could take breaks at any time. Transcripts were auto‐generated, improved for accuracy and verified by participants. Translations into English were carefully reviewed by the first author against original Spanish transcripts and audio. Anonymity was maintained in transcripts, although not feasible during group discussions. The focus group guide is in Appendix 3.

### 2.5 Analysis

The following six‐step thematic analysis approach was utilised [33]: Step 1: Become familiar with the data; Step 2: Generate initial codes; Step 3: Search for themes—revising codes around the question; Step 4: Review themes; Step 5: Define themes; and Step 6: Write up the findings. At each step, the first author undertook the following: read the transcript and listened to the audio, revised the translation, highlighted key points, made notes, inductively coded, reorganised codes, performed word searches, developed process maps, compared the maps to the ‘access to healthcare’ model, renamed codes and themes, and assessed the findings for opposite experiences. NVivo 12 (Lumivero) was used as a tool to support the analysis of responses. To ensure trustworthiness, the author had prolonged engagements with the data using reflexive journaling, theme mapping and audit trails. The UNSW researchers, who oversaw the development of all research questions, proposals and documentation, discussed and reviewed the coding tree. Draft results were interrogated and refined by C.J. and C.S. using the Levesque access model, which formed the framework for presentation of the data [26]. The quotations were chosen because they were succinct, illustrative of the point being made, and demonstrating a range of patterns in data [34].

### 2.6 Ethics

Ethics approval was granted by the University of NSW Human Research Ethics Committee. Informed consent was obtained from all participants. While anonymity in group discussions was not possible, data were anonymised in transcripts and reporting.

## 3 Results

### 3.1 Sample Characteristics

The participants were from ten Spanish‐speaking LAC in Central and South America. About half were female, most were aged between 40 and 69 years, and age of diagnosis ranged from under 30 to over 60 years. Details are provided in Table 2.

**Table 2:** Peer advocate characteristics.

Domain	Characteristic	Number
Sex	Female	12
	Male	13
Age at diagnosis (years)	Under 30	4
	30–39	5
	40–49	7
	50–59	7
	60–69	2
Years living with Parkinson's disease	0–5	6
	6–10	7
	11–19	7
	20+	5
Current age (years)	< 40	1
	40–49	7
	50–59	8
	60–69	8
	≥ 70	1
Living arrangement	Alone	3
	With partner	9
	With extended family	13
Countries of residence	Perú	6
	Argentina	4
	Mexico	4
	Chile	3
	Venezuela	2
	Colombia	2
	Ecuador	1
	El Salvador	1
	Guatemala	1
	Uruguay	1
Socio‐economics of the PD community	Evenly mixed	9
	Middle	4
	Middle to upper	1
	Lower to middle	2
	Lower	9
Education	Completed study after Secondary School	21
	Secondary schooling only	4
City or regional	Capital City	13
	Regional City	9
	Rural/village	3
Involvement with PWP (number who answered ‘yes’)	I have research experience (participant or researcher)	11
	Involved in international forums/Parkinson's‐related foundations	19
	I am a professional serving people with Parkinson's disease.	3
	I lead or educate a support group	16

**Figure 1:**
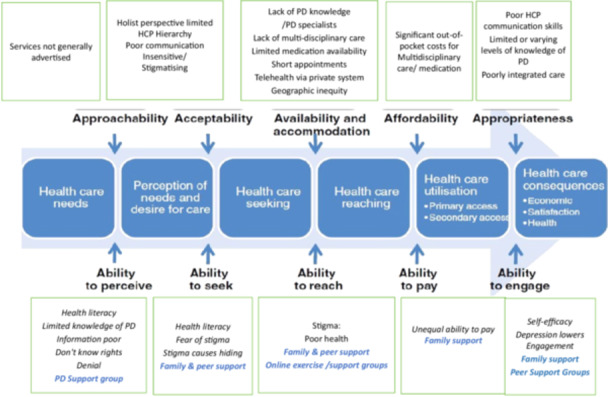
Results mapped to Levesque et al.'s conceptual framework of access to healthcare [26].

The results are summarised according to the Levesque access model [26] in Figure 1.

### 3.2 Provider Capabilities

#### 3.2.1 Approachability

Approachability refers to the visibility of relevant health services. Participants generally did not initially know where to go to access healthcare. A family in Argentina learned about a hospital when their cousin saw a promotion on television.

We found out that it is close to home, it gives monthly talks … that it gives the most complete [holistic] treatment…. So, about after 5 years [since diagnosis] we started to learn more and to connect with other people.

C7, Argentina

This was the only hospital identified as providing patient education.

#### 3.2.2 Acceptability

The acceptability of the service is based on personal, social and professional values and norms, as well as culture, gender and autonomy. Stigma‐related fear affected acceptability and impeded help seeking and participation in support groups. One participant related a story of how people contacted him after he had been on television talking about PD. They asked for advice about their PD symptoms: ‘I have a little tremor, I have this, I have that, do you think it could be Parkinson's?’ (P13, Argentina). However, they did not want to see a doctor for fear of experiencing discrimination if it was PD. Participants reported that they and other PWP they knew were afraid to meet in a support group as they did not want people to know that they had PD, to avoid being stereotyped and treated with prejudice.

The communication between doctor and patient is often culturally mediated, and one family experienced shared their experience.

There is a hierarchical relationship between the doctor, the who looks down towards the patient and relatives. How do we empower ourselves then?

C9, Peru

This approach did not help the participant engage with their care.

#### 3.2.3 Availability and Accommodation

Participants reported inadequate and unequal service availability: PD specialists, neurologists, allied health professionals with specific qualifications in PD, a lack of medicines and only short appointments could be obtained.

It took too long to get the diagnosis…. The barrier is the training of the [health] professionals in the initial symptoms of Parkinson's, like slowness, the neurologist doctor should know.

P8, Peru

While availability was generally inadequate, it was particularly poor in rural areas, and there was very limited private telehealth for those who could afford it.

Two years I was diagnosed wrongly with essential tremor by a provincial neurologist, but I noticed that it was obstructing me and that I couldn't move. I had to go to the capital, which is about 130 km from my residence…. I went to the public health service because I have limited resources … the appointment is not until February.

P19, Chile

While some participants could access services in a timely manner, others with fewer resources had less timely access.

I have more access because I have prepaid healthcare because I pay privately. I have immediate access.

P18, Columbia

The inadequate availability of a multidisciplinary and specialised PD workforce resulted in late diagnosis of PD; insufficient and inappropriate management of PD (discussed below); inadequate advanced care and medication; and substantially reduced quality of life.

One person in the study reported taking medication without a prescription as he was awaiting his formal diagnosis with the public system.

I think that there is a tremendous shortage of professionals, first because I am totally ignorant on the subject, and I am also self‐medicating without knowing what consequences it can bring me.

P25, Chile

Most participants reported a lack of Information from the doctor. Participants reported that access to medication was better in Argentina, Mexico and Chile than the other countries represented in the group.

#### 3.2.4 Affordability

Commonly, there was a system of public healthcare services that could provide free or subsidised services or alternatively people and employers made co‐contributions for health insurance or private health or a combination (requiring out‐of‐pocket payments). Neither was perfect.

The private insurance system is getting more expensive. …[but] the system that is the public system …. it gives you very basic medicine, only the basic one, …as the years go by … there are no more sophisticated, higher quality medicines.

P9, Peru

In Venezuela, Guatemala, El Salvador and Mexico, participants accessed one medication through public healthcare, and often other medications were too costly for many.

#### 3.2.5 Appropriateness

Study participants reported instances of health providers demonstrating poor communication skills, little knowledge of PD and working in isolation. Communication was described as dismissive, unhelpful and unsupportive. A participant was given her prognosis with blunt language, without an attempt to soften the hard news.

In 5 years, you will be in a wheelchair.

P18, Colombia

Such communication stigmatises the person and runs the risk of demoralising patients, erodes trust and discourages future engagement. Health professionals talked with family member rather than the patient.

I remember that the doctor sat … and he said looking at my daughter ‘How he is then?’. I didn't exist….

P9, Peru

One participant was repetitively misdiagnosed for over 15 years (P5, Mexico). When they were diagnosed, many participants reported that this consisted of little more than the phrase ‘you have Parkinson's’, followed by a prescription for medications. One participant said: ‘He sent me home without any referral’ (P17, Uruguay). She would have liked ‘an ABC guide to take home and read’ (P17, Uruguay). Information was something that participants agreed was lacking from their doctors. To access appropriate medication, one participant bought medication while on vacation in Mexico:

The variety of concentrations in medication have stopped arriving. I had the opportunity to travel to Mexico … to bring different concentrations of the medication….

P8, Peru

Availability is reducing, not improving, and this affects patients. The following participant struggles with daily activities.

I get ‘off’ periods every day throughout the day, making it very difficult for me to talk, exercise, or engage in any social activity.

P9, Peru

Thus, medication unavailability influences both quality of life and ability to engage in healthcare.

### 3.3 Consumer Abilities

#### 3.3.1 Ability to Perceive

The ability to perceive the need for healthcare was particularly challenging pre‐diagnosis. Typically, the participants first perceived that something was wrong, but lacked the health literacy of PD to find information. The little information on PD was often inaccurate. One bilingual participant stated:

When I go in Spanish groups about Parkinson's on Facebook, the level of misinformation is frightening…. I mean, if I depended on the information I receive in Parkinson's groups, in Spanish we'd be ‘screwed’ … a magical treatment … turns out to be a scammer … people fall for these … there is a very high level of misinformation in Latin America.

C22, Ecuador

Several participants reported being in denial, and two only accepted the diagnosis after seeing 14 doctors each.

#### 3.3.2 Ability to Seek

Low health literacy also influenced seeking care, with PWP reportedly lacking knowledge of what healthcare they needed and their healthcare entitlements.

I couldn't go further, I didn't know where to go, what kind of doctor to go to.

P12, Mexico

Without access to reliable information, PWP were at a loss for how to manage the disease.

Peer support groups have used online methods since Covid to try to educate PWP about PD and how to seek care, and another created a network in her country with other peers and carers. Participants became facilitators of peers ‘ability to seek’. See Table 2 which shows 16 led support groups.

Some participate in WhatsApp and then they share information…. We are trying to encourage people to be in groups, to be connected to each other and they help themselves.

P7, Argentina

Finding doctors and support groups and sharing their knowledge of their own healthcare entitlements was common among participants.

I got … a certificate of disability and in Argentina, this means medication is 100% free.

P7, Argentina

One participant explained it was an honour to support the community.

I have been enriched, I have been empowered by my peers…, so I also feel a commitment to contribute. I can help with my testimony.

P12, Mexico

This participant felt good about helping each other and empowered.

#### 3.3.3 Ability to Reach

Barriers to the ability to reach care included stigma and poor health. For all PWP, particularly those who had to travel from rural to urban locations for treatment, they required time, financial resources, adequate physical mobility, mental well‐being and support. They could be hindered by fear of embarrassment and discrimination due to PD stigma, particularly when the PD symptoms were noticeable.

When one becomes embarrassed by Parkinson's, the person isolates, and the disease progresses further…. The prejudice that others have makes people want to remain in their homes; they do not want to go out….

and if they are invited to eat or something, they give an excuse. It has also happened to me because of depression. Depression also occurs because of Parkinson's, and that makes us isolate ourselves.

P7, Argentina

To address the difficulties in reaching services during the Covid pandemic, some peer groups organised online classes, ‘physical therapy, meditation, yoga, tai chi, and painting, among others’ (P5, Mexico), which also helped address isolation.

#### 3.3.4 Ability to Pay

Some participants in the study reported that they had the ability to pay for the healthcare. However, they noted that they were not typical of PWP in their countries.

I recognize that I have enough resources or sufficient resources to access medicine … people who have fewer resources, in general, they go to the offices of the public health system and there they just give them an appointment for 6 months later.

P1, Chile

Many relied on family to assist with the costs of medicine and healthcare. Family was often mentioned as helpful in seeking healthcare like the example of a brother being helped to get a bus to Lima for treatment.

I took the courage to tell my brothers that I had this disease. So, my brother gave me a ticket to go to Lima and helped him go to clinics.

P24, Peru

This participant eventually accessed an expert in PD in Lima. He stayed in Lima for treatment and lived with members of his extended family.

#### 3.3.5 Ability to Engage

PWP were reported to be active in asking questions of health professionals, but the health professionals tended to provide little information.

I've asked questions because I have atrophies, and I don't know what they are. He just says ‘it's part of PD. He doesn't explain what that means’.

P3, El Salvador

Family members were mentioned as helpful in the process of actively engaging in care.

I'm very happy to be with my wife because she has helped me enormously. She discovered alternative treatments that the neurologist didn't know about.

P22, Ecuador

Family and peer support groups were mentioned throughout the access journey. It is noted that family support was predominantly provided by women, for example, ‘my wife’ and ‘my daughter’. A peer was also able to help connect a participant and his carer wife to a support group.

We sat and we talked. I'm grateful as she introduced us to the support group….

So, I joined a group … and people are enthusiastic. We have, let's say, about 30, but about 12‐15 of us participate. But every day we communicate through a chat, every day. It's a living group.

C9, Peru

This carer valued being in the community. Being supported helps both carers and PWP. For some participants, the facilitator of health was to proactively improve theirhealth with exercise.

The exercise, that is soccer, has served as medicine. It has kept me well … thank God my Parkinson's has not advanced. My neurologist said that I am not advancing at all.

P20, Colombia

This young‐onset participant had improved his health. Poor health had the opposite effect. Depression was repeatedly mentioned as a barrier to accessing services.

The lady is 39 years old … I ask her ‘Do you exercise?’ … but because she is very depressed, she tells me ‘no’ I explain to her, ‘but you need to exercise to be able to motivate yourself‐ if not it's a vicious circle.’

P16, Mexico

Limited access has negative consequences.

## 4 Discussion

This qualitative regional study offers a crucial insight for policymakers and healthcare providers regarding resource gaps and patient needs in Latin America, carefully mapped to Levesque et al.'s conceptual framework of access (Figure 1) [26]. The study identified the barriers and facilitators of healthcare access for PWP in 10 Spanish‐speaking LAC from the perspective of PWP who are peer advocates. Access to treatment for PWP in LAC is hindered by the lack of availability, acceptability and appropriateness of PD treatment. Many of the deficits resonate with the literature globally [5, 6, 9] and the resource constraints and stigma experienced in Africa [15, 35]. Our findings reveal the role of family and peer support, alongside increasing online access, in fostering healthcare engagement and promoting overall health and wellness.

Our study confirmed limited access to healthcare services for PD, including allied HCP services [10, 12]. HCPs lacked training in PD and often failed to recognise initial PD symptoms, resulting in diagnostic delays, particularly for women, as reported in another study [36]. Geographic, financial and digital disparities restrict access, which is not dissimilar to disparity elsewhere [6, 16, 21, 37, 38, 39]. As a result of limited access, PWP frequently experienced difficulties in accessing timely, comprehensive, evidence‐based treatment such as exercise therapies for symptom management [40, 41]. This deficit in early intervention is known to contribute to increased dependency and reduced quality of life [16, 42]. PD is inherently neurodegenerative. Kim et al. [10] observed rapid disease progression in Latin America. Recent findings of significant decline in quality of life [43] suggest a rapid pace of neurodegeneration in people with PD in the region. Healthcare training needs a focus on recognition of PD symptoms, initial diagnosis and symptom management [44].

The most surprising and disheartening finding was the prevalent accounts of dismissive communication styles employed by HCPs, which consequently led to distrust rather than much‐needed empathy and hope [19]. HCP poor communication has been previously cited in the literature as a barrier [6]. The statement ‘In 5 years *you will be in a wheelchair*’ highlights the harsh reality and the constrained expectations of some doctors in Latin America [6, 45] and undeniably contributed to patient demoralisation. This suggests the need to build sensitivity in professional communication and provide person‐centred care [45].

One of the more pervasive barriers to seeking and receiving appropriate care, reported at both system and person levels, was stigma [17]. Stigma is a complex concept that can be divided into public stigma and anticipated or self‐stigma. Public stigma comprises negative attitudes or stereotyping from others (such as health professionals) [46]. Self‐stigma exists when a person internalises this negative stereotyping [18]. Our study revealed that stigma impeded the processes of obtaining a diagnosis, receiving treatment and engaging with support groups, which can result in profound isolation for the PWP. Some literature exists on stigma in Brazil, but there is insufficient research in the Spanish‐speaking Latin American context to understand it well [21, 22, 23]. For example, elsewhere, stigma has been found to adversely affect quality of life and physical and mental health, resulting in social isolation and reluctance to seek healthcare [17]. In high‐income countries, stigma contributes to a lack of acceptance, isolation and loneliness, worsened symptom severity, and reduced quality of life for PWP [19, 22, 47, 48]. If stigma has similar impacts in Latin America, not addressing it will diminish health‐seeking and worsen both mental and physical health [15, 26, 49]. This affects quality of life and even survival in PWP in high‐ and low‐income countries [15, 50]. Stigma interventions are needed according to the World Health Organization [9, 39]. Strategies to create public awareness are needed [15, 17, 47]. The video‐based PD education campaign in Thailand, with 4.4 million YouTube views, is a good example of this [51]. The campaign received comments showing people became empathic and had a new understanding of PD [50, 51]. Ultimately, stigma is frequently rooted in misinformation about causes and must be addressed. Further research is needed to understand stigma and create inventions to address it.

This study illuminates the vital role family members, peers and support groups play in facilitating access to, and engagement with, health services for PWP. This finding is relatively undocumented in Latin America but resonates with research from Africa, where peer support groups provided a source of education, social support and legitimacy that participants perceived as largely absent from formal healthcare systems [35]. While most study participants were highly engaged, they reported that most PWP, whom they knew, were less engaged. One participant shared her testimony ‘*to encourage others’* because she felt ‘*empowered by her peers*’, reflecting how such support fostered psychological strength and social cohesion [52]. Similarly, a UK‐based study highlighted that peer narratives supported individuals in adapting to lifestyle changes, including engagement with exercise [53]. Family members helped PWP to access care and provided emotional support, aligning with findings by Zhu et al. [54]. Our findings suggest carers enjoy support and confirm the need for self‐care for carers [55, 56]. Across diverse LAC, ‘social support’ has been consistently reported as the most important factor for ‘living well’ with PD [57]. Our study confirmed the importance of social support and promoting and supporting peer groups [19, 58].

The Covid‐19 pandemic unexpectedly accelerated the adoption of online platforms, enabling PWP to access educational and peer support forums from home [4, 35, 59]. In this study, 19 of the 25 participants engaged with such online forums (see Table 2), which facilitated disease‐specific knowledge acquisitions, opportunities to ask questions, reduced stigma and barriers to exercise. Previous research similarly confirms that online group participation can reduce isolation and improve quality of life among PWP in Africa [60]. Despite improved access, beyond webinars, the study found that online content poses challenges for PWP with low health literacy, who may struggle to identify trustworthy sources [4]. This underscores the need for credible self‐management resources from a trusted source and basic health literacy training to support informed online engagement [4]. Online solutions could be further utilised for training health professionals or for online appointments, telehealth, albeit with a need for HCP upskilling in online PD management [61].

Self‐management interventions, widely adopted in high‐income countries, support PWP and carers in building the knowledge and skills necessary to manage symptoms, medication and psychosocial well‐being [62, 63]. These approaches typically include structured resource kits (in print, digital or app‐based formats) and promote patient activation through education, emotional support, exercise and peer engagement [64]. Self‐management can reduce hospitalisations and improve outcomes in chronic disease populations [47, 64, 65]. Self‐management interventions remain largely absent across LAC. Health systems and NGOs should invest in the development of a self‐management programme based on a core resource kit that can be adapted. Co‐designed strategies could ensure culturally relevant materials and Latin American examples of success, like the Saturday in Motion project in Colombia [66]. Another example of a project is A Movement Disorder Society‐sponsored partnership between the University of Rochester and the hospital in regional Peru (Arequipa), which has demonstrated the feasibility of international partnerships to build capacity and of HCP and PWP and could be adapted to teach PD self‐management skills and improve patient outcomes [67]. Replicating such models in other underserved regions of Latin America could accelerate the dissemination of evidence‐based care and expand access to person‐centred support, which has been done many times before [2, 68, 69, 70, 71]. It is prudent to carefully consider the research to evaluate how these partnerships influence care delivery and whether tailored self‐management interventions can improve health equity in LAC contexts.

## 5 Limitations

Due to the requirements for written consent, a stable internet connection, computer literacy, and work as a peer advocate or educator, study participants tended to be more educated and financially resourced than the population of PWP in LAC. Participants were younger than most PWP, as PD is most frequent in people over 60 years of age; however, only 9 of the 25 participants were in that age group. While the group was not typical of PWP in their country, they brought the experience and stories of PWP with whom they worked and who they taught, thus providing a wider diversity of experiences than their own. Some participants were very eloquent, and their words featured more in the findings than others. However, their views were generally reflective of the views of the group. These findings are not necessarily representative of all experiences across the countries included, but point to common patterns of service under‐provision and lack of PD awareness and education amongst HCPs across the countries.

## 6 Recommendations

All recommendations should be enacted in Partnership with Parkinson's experts, HCP and PWP and carers:

1. **Stigma and anti‐stigma interventions:**
  Research is needed to further understand stigma and barriers to healthcare to create interventions tailored to each country in Latin America. It is recommended that interventions adopt a multilevel educational approach with patients, family, community, support groups, health professionals and media campaigns for the public.
2. **Creation or adaption of a self‐management toolkit** to address the deficit of information from reliable sources and to provide HCP and patients and family a tool where they can be one the same page. Training is needed for HCPs and peer leaders, PWP and family in self‐management.
3. **Training of HCPs** in person‐centred care and focused on symptoms of Parkinson's, diagnosis and self‐management as per the self‐management toolkit.
4. **Global collaboration** and partnerships between universities and hospitals could improve PD management capacity and **pilot a self‐management approach** in regional cities of LAC which would be highly advantageous.

## 7 Conclusion

This study makes a significant contribution to the field by shifting the lens from provider‐centric views to the lived realities of PWP in Latin America—voices that have been largely absent in the research to date. It identifies systemic limitations in diagnostic capacity and care quality, exacerbated by insufficient HCP training and stigma, a deeply embedded, culturally shaped barrier to care that needs further research to identify interventions that can remove stigma. Peers, family and support groups are facilitating access to healthcare, but a self‐management framework would be useful to strengthen the roles of family and peers further. Funding bodies, policymakers, HCPs and peer advocates each have a critical role to play in collaborative efforts to fund, prioritise and co‐design and implement a self‐management toolkit. Working together is the best way to address the barriers and celebrate the strengths and self‐management potential of the PD community in Latin America.

## Author Contributions

**Christine Jeyachandran:** lead author, study design, data collection, analysis, interpretation, writing, editing. **Catherine Spooner:** academic supervisor, study design, data analysis, writing. **Ana Margarita Rodriguez Salgado:** focus group lead, data collection, analysis, interpretation, reviewing. **Matthew Prina:** chief investigator of PINNACLE Team, Michael J. Fox Grant, study design, writing, editing. **Joel Rhee:** academic supervisor, analysis, interpretation, writing. **Jorge Jesus Llibre‐Guerra:** grant application, study design, editing. **Dani Kim:** study design, editing. **Juan J. Llibre‐Rodriguez:** study design. **Mark F. Harris:** primary academic supervisor, study design, analysis and interpretation, writing.

## Disclosure

The funder had no role in the study design, data collection and analysis, decision to publish, or preparation of the manuscript.

## Ethics Statement

The project was approved by the University of New South Wales (UNSW) Human Research Executive Committee on 8 June 2023 (identification number HC230231).

## Consent

All participants provided written informed consent.

## Conflicts of Interest

The authors declare no conflicts of interest.

## Supporting information

Appendix_1_Call_Script.

Appendix_2_Focus_Group_Composition.

Appendix_3_Focus_Group_Guide.

## Data Availability

Code lists are available upon request from the corresponding author. Our Ethics Committee approval does not allow the provision of research data to external researchers.
