# Supplementary material for: Barriers and Facilitators to Accessing Healthcare for People With Parkinson's Disease in Latin America: A Qualitative Study
Source: Health Expect. 2025 Aug 13;28(4):e70380. doi: 10.1111/hex.70380 (PMC12344579; doi:10.1111/hex.70380)
Supplement: Supplementary file 1 — Appendix_1_Call_Script. [file HEX-28-e70380-s002.docx]

**Appendix 1.** Call Script

The purpose of the call is to check suitability for the focus group study.

A scripted call is designed to

- verbally confirm EOI (Expression of Interest) details in a way that builds trust.
- give us an opportunity to explain the focus group and answer questions.
- an opportunity to see if their voice can be understood (Voice can be weakened in Parkinson’s Disease and a care giver support may be needed.
- check if they are fluent in Spanish or have carer support for this.
- check cognitive capacity, and check if carer involvement is planned or needed
- build trust with participants.

No undue pressure will be placed on the participant.

**If concern arises, we will contact a** **carer.**

Potential concerns:

1. the person does not remember the research or filling in the EOI,
2. the verbal identity is inconsistent with EOI,
3. PWP (People with Parkinson) confusion or not grasping details of focus groups or technical issue that is unresolved (cannot do a Teams call)

If there is a concern, we will ask to consult a carer/ family member concerning the research. They can support the PWP’s involvement, and they can attend with them if they wish. If this is not desired or possible, we will respect the participant's desire not to have us talk to a family member.

The PWP will decide if they are involved. The carer EOI will give the carer a chance to express concerns or be present for the focus groups if the PWP agrees. If a concern is flagged and secondary consent cannot be gained, the participant will not be advanced to the focus groups.

**SCRIPT**

1. Hi _____________, I am Christine Jeyachandran from Alianza and the
   “access to healthcare for people with Parkinson’s in Latin America” research project
2. Pause to see if they recognize you.
3. Thank you for filling the EOI, It is a pleasure meet you ________(NAME).

I have read your Expression of Interest Form and I thought I would ask you to tell me about yourself in your own words. ‘Who is _____?”

(Ask for Participant and have them confirm their identity ________)

Great. Nice to get to know you a bit.

I want to check that you are eligible to take part in the FOCUS Group Study

Let me confirm Check Eligibility/ Exclusion Criteria – If ineligible explain this and end call, or if eligible continue:

**Participants in this study need to meet the following Inclusion criteria. Respond yes or no to the following questions:**

A peer advocate is a PWP engaged in peer support/ education and advocacy in Latin America. This could include being a member/leader in a support group/ community or a workers/ paid or volunteer in a government, non-government or community organization that serves PWP. I am a Parkinson’s peer advocate? Yes/ No

I have Parkinson’s Disease which has been diagnosed by neurologist. Yes/ No

I have experience living with Parkinson’s disease in Latin America. Yes/ No

I can converse in Spanish. Yes/ No

I am over 18 years of age. Yes/ No

I can participate in online meetings (possibly with career help). Yes/ No

I can understand and respond to questions. Yes/ No

OK you are eligible, or you are Confirm if they want to be involved

or

Sorry you do not quite fit the criteria for this study at the moment. Thank you for your time. (End call)

If eligible:

We would like to see if you are suitable for a focus group, but we need to check a few things first.

Do you know how to do an online phone call or meeting (e.g., on Zoom or Microsoft TEAMS?)

We are having a practice session at X time…?

Here is the link to connect.

Would you prefer a family member or carer to help you?

You are welcome to have a carer present to comment or participate too.

Will you have a care giver present or not?

Insert reasons for carer again

If not provided earlier, ask for Carer phone what their phone is/ email so they can complete this form.

Great.

At the practice call we will confirm your focus group session time:

Do you have any questions?

Thank you for your time.
