# Supplementary material for: Barriers and Facilitators to Accessing Healthcare for People With Parkinson's Disease in Latin America: A Qualitative Study
Source: Health Expect. 2025 Aug 13;28(4):e70380. doi: 10.1111/hex.70380 (PMC12344579; doi:10.1111/hex.70380)
Supplement: Supplementary file 2 — Appendix_2_Focus_Group_Composition. [file HEX-28-e70380-s001.docx]

| **Appendix 2: Focus Group Composition** | | | | |  |
| --- | --- | --- | --- | --- | --- |
| Composition was based on availability because we were working across 5 time zones.  The P stands person and they each received a number in order of focus group talking. The Carer has the same number as PWP. To preserve anonymity, limited participant data are shown.  Minimum criteria for study composition was approximately half from each gender, half were from a city vs a regional area/ village and a variety of countries (variety is other characteristics). We monitored the composition as they enrolled to ensure a variety of participants. The final group was recruited via follow up email to address the deficit in from men, participants from countries with only 1 or less (at that point) or (Ecuador Chile Colombia), and regional/ rural areas. | | | | | |
| **Group** | **Gender** | **Code** | **Person with Parkinson's or carer** | | **Country** |
| **Group A - Ages 44-70, 7-25 years with PD** | | | |  |  |
| Group A | Male | P1 | Person with Parkinson's | | Chile |
| Group A | Female | P2 | Person with Parkinson's | | Argentina |
| Group A | Female | P3 | Person with Parkinson's | | EL Salvador |
| Group A | Female | P4 | Person with Parkinson's | | Venezuela |
| Group A | Female | P5 | Person with Parkinson's | | Mexico |
| Group A | Male | P6 | Person with Parkinson's | | Peru |
| **Group B Ages 50-70, 8-30 years** | | | |  |  |
| Group B | Female | P7 | Person with Parkinson's | | Argentina |
| Group B | Female | C7 | Carer |  | Venezuela |
| Group B | Female | P8 | Person with Parkinson's | | Peru |
| Group B | Male | P9 | Person with Parkinson's | | Peru |
| Group B | Female | C9 | Carer |  | Peru |
| Group B | Male | P10 | Person with Parkinson's | | Guatemala |
| Group B | Female | P11 | Person with Parkinson's /Carer | | Mexico |
| **Group C, Ages 51-72 , 5-34 years with PD** | | | |  |  |
| Group C | Female | P12 | Person with Parkinson's | | Mexico |
| Group C | Male | P13 | Person with Parkinson's | | Argentina |
| Group C | Male | P14 | Person with Parkinson's | | Venezuela |
| Group C | Female | C14 | Carer |  | Venezuela |
| Group C | Female | P15 | Person with Parkinson's | | Argentina |
| Group C | Female | P16 | Person with Parkinson's | | Mexico |
| Group C | Female | P17 | Person with Parkinson's | | Uruguay |
| **Group D, 40-70, 1-22 years with PD** | | | |  |  |
| Group D | Female | P18 | Person with Parkinson's /Carer | | Colombia |
| Group D | Male | P29 | Person with Parkinson's | | Chile |
| Group D | Male | P20 | Person with Parkinson's | | Colombia |
| Group D | Male | P21 | Person with Parkinson's | | Peru |
| Group D | Male | P22 | Person with Parkinson's | | Ecuador |
| Group D | Female | c22 | Carer |  | Ecuador |
| Group D | Male | P23 | Person with Parkinson's | | Peru |
| Group D | Male | P24 | Person with Parkinson's | | Peru |
| Group D | Male | P25 | Person with Parkinson's | | Chile |
|  |  |  |  | |  |
