# Supplementary material for: Barriers and Facilitators to Accessing Healthcare for People With Parkinson's Disease in Latin America: A Qualitative Study
Source: Health Expect. 2025 Aug 13;28(4):e70380. doi: 10.1111/hex.70380 (PMC12344579; doi:10.1111/hex.70380)
Supplement: Supplementary file 3 — Appendix_3_Focus_Group_Guide. [file HEX-28-e70380-s003.docx]

Appendix 3: Focus Group guide

## Introduction - Research and Researcher

Introductions of researcher including location, experience and interest in PD and role.

- Thank participants for coming

- explain how the session will work (an hour and half).

- set ground rules around participation that asks them to respect other participants and confidentiality etc

- Remind participants they are consenting to being involved and recorded and why it is being recorded.

- Invite feedback from all participants. Note: The first focus group will be a pilot focus group and feedback and reflection from the researchers and participants will be used to improve the latter groups and refine questions or processes.

- We will ask if they have any questions before we start.

## Responding to Questions

*Explain that we want to hear about the experiences of their communities of PD in Latin America*

Our time will be divided between the three questions. There will be about 20-25 minutes for each of them. In your responses, please

- give specific examples from your community and
- explain who you are talking about in your community, any relevant context specifics and
- we may probe you for more details about was this one person’s experience or a broader community experience and
- how do you know this information and other clarifying questions like what, where, when and who?

That said we need to ensure responses are succinct and allow for others to share the experiences of their community too.

## **The 3 Areas of study:**

**This project aims to identify the barriers and facilitators to access to healthcare for people with Parkinson’s Disease (PWP) in Latin America**

**The research questions are from the perspective of peer advocates. They are:**

**1. What are the unmet healthcare needs of Latin American PWP (LAPWP)?**

**2. What are the barriers and facilitators to access to healthcare for LAPWP?**

**3. What are the priorities for further research?**

### Instructions

Participants and carers: Before giving your information, please introduce yourself with your name, where you live, a brief comment about your diagnosis, and what peer advocacy work you do?

**A: Treatment and unmet needs [X minutes each participant, total Y minutes]**

1. What Parkinson’s healthcare did you receive when you were first diagnosed, and what thereafter? (brief)

2. Tell us a specific story of someone from the community you know and how their experience was the same or different from yours. What factors stood out? These stories will help identify some of the main pathways for management of Parkinson’s in your country.

3. What are the unmet healthcare needs for Parkinson’s in your country?

**B. Barriers and facilitators to access accessing the pathways of care for Parkinson’s in your country. [X minutes each participant, total Y minutes]**

**Explanation:** Barriers are things that prevent PWP from receiving the healthcare they need for their health and wellbeing, or make it harder to receive healthcare, or affect the quality/usefulness of the healthcare received. Facilitators can be the opposite - make it easier to receive care or help to receive care.

**Questions:**

1. Tell me a story or example of a barrier/ facilitator in your country.

2. What are the most common barriers /facilitators?

3. Are the barriers and facilitators institutional and/or personal?

4. What helps people get access to healthcare treatment in your country? (facilitators)

5. What prevents people getting access to health care in your country? (barriers)

**C. Considering the barriers and facilitators to access healthcare for PWP in Latin America, what are the priorities for further research?**

Our aim is to define and **prioritise a shortlist of the top unmet needs** in healthcare for Parkinson’s in your country. A problem needs solutions or ideas of how to address the unmet need. How should these priorities be addressed? Define which factors should inform the selection of an unmet need as a priority.

Factors to consider in Prioritisation of needs in view of barriers and facilitators:

- the size of the problem, i.e... most people affected.

- the evidence-based response is effective solution available.

- feasibility of solutions,

- likely impacts, (broad impact)

- fast impact

- cost effectiveness (cost of implementation)

- Ease of implementation (understanding and less barriers)

- ability to replicate.

- Cornerstone characteristics

What are the most pressing unmet needs in terms of how healthcare Parkinson’s in your community/ country?

We will Ideas from everyone and a why it is important, then return to consider each one and consider factors affecting each.

D. **Probes:**

Who should decide between these priorities? What role should PWPD, and carers play?

Is the solution more research or a clear recommendation that does not require further proof/ investigation?

E. **Vote on the top three priority themes for research/solutions
 (with your reasons) as a group and by country.**

Thank-you for your time. If you have any questions or feedback for us, please email Christine.

After the call – Participants can stay longer and debrief with first author about the experience and give feedback.

In addition, a breakout room will be enabled after the session for anyone who wants to discuss with a psychologist, NAME DELETED, who will be available to talk to anyone in distress or who has concerns.
